# ﻿Notes on the genus *Elegansovella* Hirschmann, 1989 (Acari, Mesostigmata, Urodinychidae)

**DOI:** 10.3897/zookeys.1205.125164

**Published:** 2024-06-19

**Authors:** Jenő Kontschán, Sergey G. Ermilov, Stefan Friedrich

**Affiliations:** 1 Plant Protection Institute, HUN-REN Centre for Agricultural Research, Budapest, Hungary Plant Protection Institute, HUN-REN Centre for Agricultural Research Budapest Hungary; 2 Department of Plant Sciences, Albert Kázmér Faculty of Mosonmagyaróvár, Széchenyi István University, Mosonmagyaróvár, Hungary Széchenyi István University Mosonmagyaróvár Hungary; 3 Tyumen State University, Institute of Environmental and Agricultural Biology (X-BIO), Tyumen, Russia Tyumen State University Tyumen Russia; 4 Ludwig-Maximilians-University Munich, Faculty of Biology, Biocenter LMU, Planegg-Martinsried, Munnich, Germany Ludwig-Maximilians-University Munich Munnich Germany

**Keywords:** Morphology, new combination, new genus, new species, Oriental and Neotropical regions, taxonomy, Uropodina

## Abstract

The genus *Elegansovella* Hirschmann, 1989 (Mesostigmata: Uropodina: Urodinychidae) is resurrected for species of the *Uroobovellaelegans*-group. This genus differs from the other taxa of *Uroobovella* Berlese, 1903 sensu lato based on the shape of the idiosoma and the caudal and dorsal setae. Three species from the *Uroobovellaelegans*-group are transferred to the genus *Elegansovella*, as *E.pectintata* (Hirschmann, 1973), **comb. nov.**, *E.pectinatasimilis* (Hiramatsu, 1980), **comb. nov.** and *E.serangensis* (Hiramatsu, 1980), **comb. nov.** The other seven species from this species group are transferred to *Monstrobovella***gen. nov.**, as *M.crustosa* (Vitzthum, 1926), **comb. nov.**, *M.enodis* (Hiramatsu, 1985), **comb. nov.**, *M.faceta* (Hiramatsu & Hirschmann, 1978), **comb. nov.**, *M.facetaoides* (Hiramatsu & Hirschmann, 1978), **comb. nov.**, *M.imadatei* (Hiramatsu, 1980), **comb. nov.**, *M.incerta* (Hiramatsu & Hirschmann, 1978), **comb. nov.** and *M.incertaoides* (Hiramatsu & Hirschmann, 1978), **comb. nov.** The new genus differs from *Elegansovella* by the shape of the idiosoma and the shape of marginal and dorsal setae. Six *Monstrobovella* species occur in the Oriental Realm and only one species is known from the Neotropical region. The present paper contains the description of a second Neotropical species of *Monstrobovella* (*M.mancocapaci***sp. nov.**) which was found in Peru. The new species differs from its Neotropical congener in the dorsal and marginal setation.

## ﻿Introduction

Giovanni Canestrini (1835–1900), the internationally noted acarologist, who also investigated the mites of the Bismarck Archipelago, discovered and described a new and unusual Uropodina mite: *Deraiophoruselegans* Canestrini, 1897. Werner Hirschmann, the noted Uropodina researcher, described a new species, *Deraiophoruspectinatus* Hirschmann, 1973 from New Guinea, which was later transferred (Hiramatsu and Hirschmann 1978) to the large catch-all genus *Uroobovella* Berlese, 1903, as *U.pectinatus* (Hirschmann 1973). Hirschmann (1973) synonymized *D.elegans* and *D.pectinatus*, and later he mentioned them only in terms of their synonymised name. Hirschmann (1989) revised this synonymy and presented both species again as two different taxa. Furthermore, Hirschmann and his co-worker Nabo Hiramatsu described seven new species from Indonesia and New Guinea (Hiramatsu and Hirschmann 1978; Hiramatsu 1980, 1985) and one new species from Ecuador (Hiramatsu and Hirschmann 1978). Additionally, Trachyuropoda (Dinychura) crustosa Vitzthum, 1926 was also transferred to *Uroobovella*. The systematic position of these species was not clear in Hirschmann’s specific Gangsystematik system, therefore, he established a new species group for these eleven species (the *Uroobovellaelegans*-group) and established in the same work a new genus, *Elegansovella* Hirschmann, 1989, for these species too (Hirschmann 1989).

An intensive acarological survey has been conducted on Peruvian mites for several years. Within this study, numerous species of Uropodina have also been discovered and described from Peru (Kontschán and Friedrich 2017, 2018, 2020a, 2020b; Błoszyk et al. 2019; Kontschán et al. 2023). In the current investigation of unidentified Uropodina species, a new species from the *Uroobovellaelegans*-group was discovered in Peru. Therefore, we started studying this species group and recognised some problems as the *Uroobovellaelegans*-group contains two species assemblages based on their morphology. The first corresponds to the genus *Elegansovella*, which has four species; the other seven belong to a new, previously undescribed genus.

## ﻿Materials and methods

Specimens of the herein presented species were found at ACP Panguana in the Peruvian Amazonia in Peru. All specimens investigated were cleared in lactic acid for a week and were then placed on half-covered well slides and examined using a Leica 1000 microscope with a drawing tube. All specimens are stored in ethanol and deposited in the Museo de Historia Natural, Universidad Nacional Mayor de San Marcos, Lima, Peru (MUSM) and SNSB-Zoologische Staatssammlung, Munich (ZSM).

Abbreviations: setae and pores: ***h*** = hypostomal seta, ***st*** = sternal seta. All measurements and the scales in the figures are given in micrometres (μm).

## ﻿Taxonomy

### ﻿Family Urodinychidae

Taxon classificationAnimaliaMesostigmataUrodinychidae

Berlese, 1917

BAA23FC2-8AE2-51B5-B9B9-1BB11EBEF99A

#### Remarks.

We provisionally retain the position of the genus *Elegansovella* in the family Urodinychidae on the basis of the following characters: setae *h1* long; chelicerae with internal sclerotized node and without mushroom- or flower-shaped sensory organ on fixed digit; corniculi smooth apically. However, all taxa of this family merit revision.

### ﻿Genus Elegansovella

Taxon classificationAnimaliaMesostigmataUrodinychidae

Hirschmann, 1989

3F6A5D7F-5AE1-5FE5-AD06-9F60BC65058D

Elegansovella Hirschmann, 1989: 102.
Uroobovella elegans -group Hirschmann, 1989: 97.
Elegansovella —Halliday 2015: 113.

#### Type species.

*Deraiophoruselegans* Canestrini, 1897, by original designation.

#### Diagnosis.

Idiosoma oval with vortex. One pair of anterolateral prolongations presented on idiosomal margin. Dorsal and marginal shields fused on anterior part of idiosoma. Dorsal setae tree-like, with a short stem and many long cross-bars. Three or four pairs of long and pilose setae situated close to posterior margin of dorsal shield. Marginal setae long and marginally pilose, situated on long marginal prolongations. Shape of female genital shield oval or scutiform, its surface smooth and situated between coxae II–IV. Male genital shield circular and situated between coxae IV. Ventral setae pilose. Tritosternum with vase-like base, its laciniae divided into three branches. Corniculi smooth, horn-like, internal malae serrate. Setae *h1* smooth and longer than other hypostomal setae, *h2*–*h4* serrate, but *h4* divided into two or three serrate branches. Pedofossae present, but without separated furrow for tarsi IV. Prestigmatid part of peritreme with five bends. Epistome serrate. Chelicerae with internal sclerotized node, fixed digit longer than movable digit. Movable digit with one or two large central teeth.

##### ﻿List of the known species

### ﻿ Elegansovella elegans

Taxon classificationAnimaliaMesostigmataUrodinychidae

(G. Canestrini, 1897)

1BE36668-08AF-5CE1-A269-D2F1888A8BDF

Deraiophorus elegans G. Canestrini, 1897: 462.
Uroobovella elegans —Hiramatsu and Hirschmann 1978: 79.
Deraiophorus pectinatus Hirschmann, 1973: 77–78.
Uroobovella pectinata —Hiramatsu and Hirschmann 1978: 85.

#### Occurrence and habitat.

This species was collected in Bismarck Archipelago and New Guinea (Hirschmann 1989).

#### Note.

Hirschmann (1973) mentioned *D.elegans* as the synonym of *D.pectinata*. Later, he (Hirschmann 1989) separated these two species again based on the shape of the basis of the epistome and the width of the peritreme. In our opinion, these differences are too weak and not sufficient to separate the two species. Furthermore, both species were collected in the same region. Thus, we agree with Hirschmann’s (1973) opinion that *D.elegans* is the junior synonym of *D.pectinata*.

### ﻿ Elegansovella pectinatasimilis

Taxon classificationAnimaliaMesostigmataUrodinychidae

(Hiramatsu, 1980) comb. nov.

4D72984E-6CBA-5A20-BB69-80FCB9FF5781

Uroobovella pectinatasimilis Hiramatsu, 1980: 49.

#### Occurrence and habitat.

This species was found in a forest habitat, in Indonesia (Hiramatsu 1980).

### ﻿ Elegansovella serangensis

Taxon classificationAnimaliaMesostigmataUrodinychidae

(Hiramatsu, 1980) comb. nov.

320673AB-3A8F-5BE3-A132-3BFEA45865BF

Uroobovella serangensis Hiramatsu, 1980: 49.

#### Occurrence and habitat.

This species was also reported from a forest habitat, in Indonesia (Hiramatsu 1980).

### ﻿Key to species of the genus *Elegansovella*

**Table:** 

1	Setae close to caudal margin of dorsal shield marginally serrate, vertex longer than wide	***E.elegans***
–	Setae close to caudal margin of dorsal shield marginally pilose, vertex shorter than wide	**2**
2	Genital shield of female oval, ventral setae with a short stem and many long cross-bars	***E.pectinatasimilis***
–	Genital shield of female scutiform, ventral setae wide and apically pilose	***E.serangensis***

### ﻿Genus Monstrobovella gen. nov.

Taxon classificationAnimaliaMesostigmataUrodinychidae

F5F2E606-01FF-5326-9A70-C01480683902

https://zoobank.org/24822A35-6D59-444B-A7C4-ABDA2CBFE789

#### Diagnosis.

Idiosoma pentagonal without vortex. Dorsal and marginal shields fused on anterior part of idiosoma. Dorsal setae T-shaped or setiform. Several longer or wider setae situated close to posterior margin of dorsal shield. Marginal setae very wide, phylliform and marginally serrate. Shape of female genital shield linguli- or scutiform, its surface smooth and situated between coxae II–IV. Male genital shield circular and situated between coxae IV. Ventral setae T-shaped, V-shaped or setiform. Pedofossae present, but without separated furrow for tarsi IV. Prestigmatid part of peritreme with two bends. Tritosternum with vase-like base, its laciniae divided into three branches. Corniculi smooth, horn-like, internal malae gently serrate. Setae h1 smooth and longer than other hypostomal setae, *h2* smooth or serrate, *h3* serrate, *h4* divided into two or three serrate branches. Epistome serrate. Chelicerae with internal sclerotized node, fixed digit longer than movable digit. Movable digit with one or two large central teeth.

#### Type species.

*Uroobovellafaceta* Hiramatsu & Hirschmann, 1978.

#### Etymology.

The name of the new genus refers to a combination of the words monster (based on extreme morphology) and *Uroobovella*.

#### Gender.

Feminine.

#### Notes.

**Figure 1.**
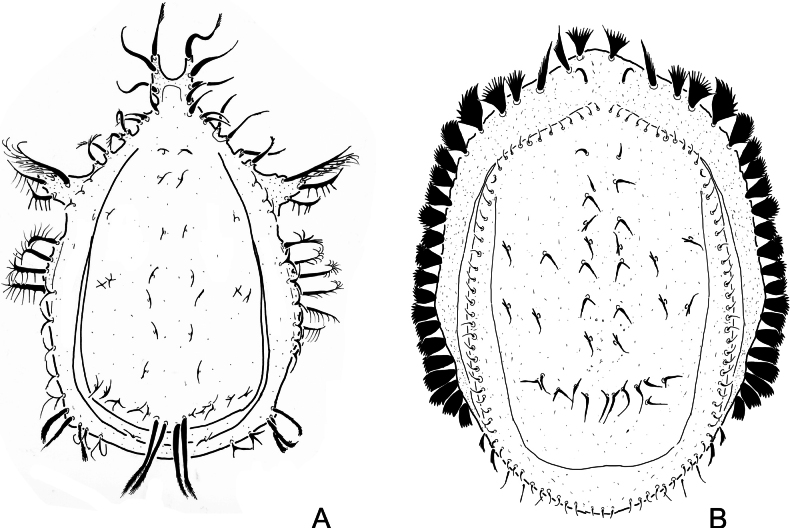
Schematic illustration of the genera *Elegansovella* (**A**) and *Monstrobovella* (**B**).

The new genus differs in many characters from *Elegansovella.* The distinguishing characteristics are summarized in Table 1 and a schematic illustration of the two genera is presented in Fig. 1.

**Table 1.** Most important differences between *Elegansovella* and *Monstrobovella* genera.

	*Elegansovella*	*Monstrobovella*
Shape of idiosoma	oval	pentagonal
Vertex	present	absent
Marginal setae	long and pilose	phylliform and apically serrate
Setae on caudal part of dorsal shield	extremely long (four-six times longer than dorsal setae)	not very long (two times longer than dorsal setae)
Anterolateral prolognation	present	absent
Peritreme	with several bends	with two bends

##### ﻿List of the known species

### ﻿ Monstrobovella crustosa

Taxon classificationAnimaliaMesostigmataUrodinychidae

(Vitzthum, 1926) comb. nov.

63147C9B-4541-5F46-87C9-023A45288E66

Trachyuropoda (Dinychura) crustosa Vitzthum, 1926: 112–117.
Uroobovella crustosa –Hirschmann and Zirngiebl-Nicol 1962: 59, 72.

#### Occurrence and habitat.

This species was found in soil, in Malaysia (Vitzthum 1926).

### ﻿ Monstrobovella enodis

Taxon classificationAnimaliaMesostigmataUrodinychidae

(Hiramatsu, 1985) comb. nov.

C48F800A-FA94-5794-9619-3B3944625817

Uroobovella enodis Hiramatsu, 1985: 5–7.

#### Occurrence and habitat.

This species was collected in soil, in Borneo (Malaysia) (Hiramatsu 1985).

### ﻿ Monstrobovella faceta

Taxon classificationAnimaliaMesostigmataUrodinychidae

(Hiramatsu & Hirschmann, 1978) comb. nov.

02FDAB45-2D97-5A15-BA8C-B5239733DCE7

Uroobovella faceta Hiramatsu & Hirschmann, 1978: 74–75.
Uroobovella faceta —Kontschán 2016: 95.

#### Occurrence and habitat.

This species was collected in leaf litter in natural and agricultural areas, in Ecuador (Hiramatsu and Hirschmann 1978; Kontschán 2016).

### ﻿ Monstrobovella facetaoides

Taxon classificationAnimaliaMesostigmataUrodinychidae

(Hiramatsu & Hirschmann, 1978) comb. nov.

107B3DBA-64D8-5877-960A-030108A82E0F

Uroobovella facetaoides Hiramatsu & Hirschmann, 1978: 76.

#### Occurrence and habitat.

This species was collected in New Guinea, its habitat is unknown. (Hiramatsu and Hirschmann 1978).

### ﻿ Monstrobovella imadatei

Taxon classificationAnimaliaMesostigmataUrodinychidae

(Hiramatsu, 1980) comb. nov.

A60997AC-6E8D-5318-A5AC-A04D8965C0B3

Uroobovella imadatei Hiramatsu, 1980: 48–49.

#### Occurrence and habitat.

This species was collected in a forest, in Indonesia (Hiramatsu 1980).

### ﻿ Monstrobovella incerta

Taxon classificationAnimaliaMesostigmataUrodinychidae

(Hiramatsu & Hirschmann, 1978) comb. nov.

699D3722-8EAE-5632-A095-7908B64033C9

Uroobovella incerta Hiramatsu & Hirschmann, 1978: 76–77.

#### Occurrence and habitat.

This species was found in New Guinea, its habitat is unknown. (Hiramatsu and Hirschmann 1978).

### ﻿ Monstrobovella incertaoides

Taxon classificationAnimaliaMesostigmataUrodinychidae

(Hiramatsu & Hirschmann, 1978)

430FCF52-E930-597F-9130-1A865A9619B7

Uroobovella incertaoides Hiramatsu & Hirschmann, 1978: 77.

#### Occurrence and habitat.

This species was collected in New Guinea, its habitat is unknown. (Hiramatsu and Hirschmann 1978).

### ﻿ Monstrobovella mancocapaci sp. nov.

Taxon classificationAnimaliaMesostigmataUrodinychidae

78BA1F4D-8B8D-5316-BE4C-FA684742A9B5

https://zoobank.org/797AB257-D04B-4A76-BAF6-D7C20FA770D5

Figs 2 , 3 , 4

#### Material examined.

***Holotype*.** Female. One female. Peru, Huánuco Department, Yuyapichis, ACP Panguana, 9°37'S, 74°56'W, 230 m a.s.l., Winkler extraction, 20 September to 07 October 2013, leg. S. Friedrich & F. Wachtel. ***Paratypes*.** One female and one male. Locality and date same as for holotype. Holotype and two female and four male paratypes deposited in MUSM, other paratypes in the ZSM.

#### Diagnosis.

Dorsal and ventral idiosoma without sculptural pattern, only some small oval pits situated on posterocentral area of dorsal shield. Dorsal setae smooth and robust, but several marginally pilose setae situated close to posterior margin of dorsal shield. Marginal setae very wide, phylliform and marginally serrate. Ventral setae smooth and setiform. Shape of female genital shield linguliform. Male genital shield oval and situated between coxae IV.

#### Description.

**Female** (*N* = 2). Shape of idiosoma pentagonal, colour yellowish brown, flat. Length of idiosoma 630, width at level of coxae IV 540.

**Figure 2.**
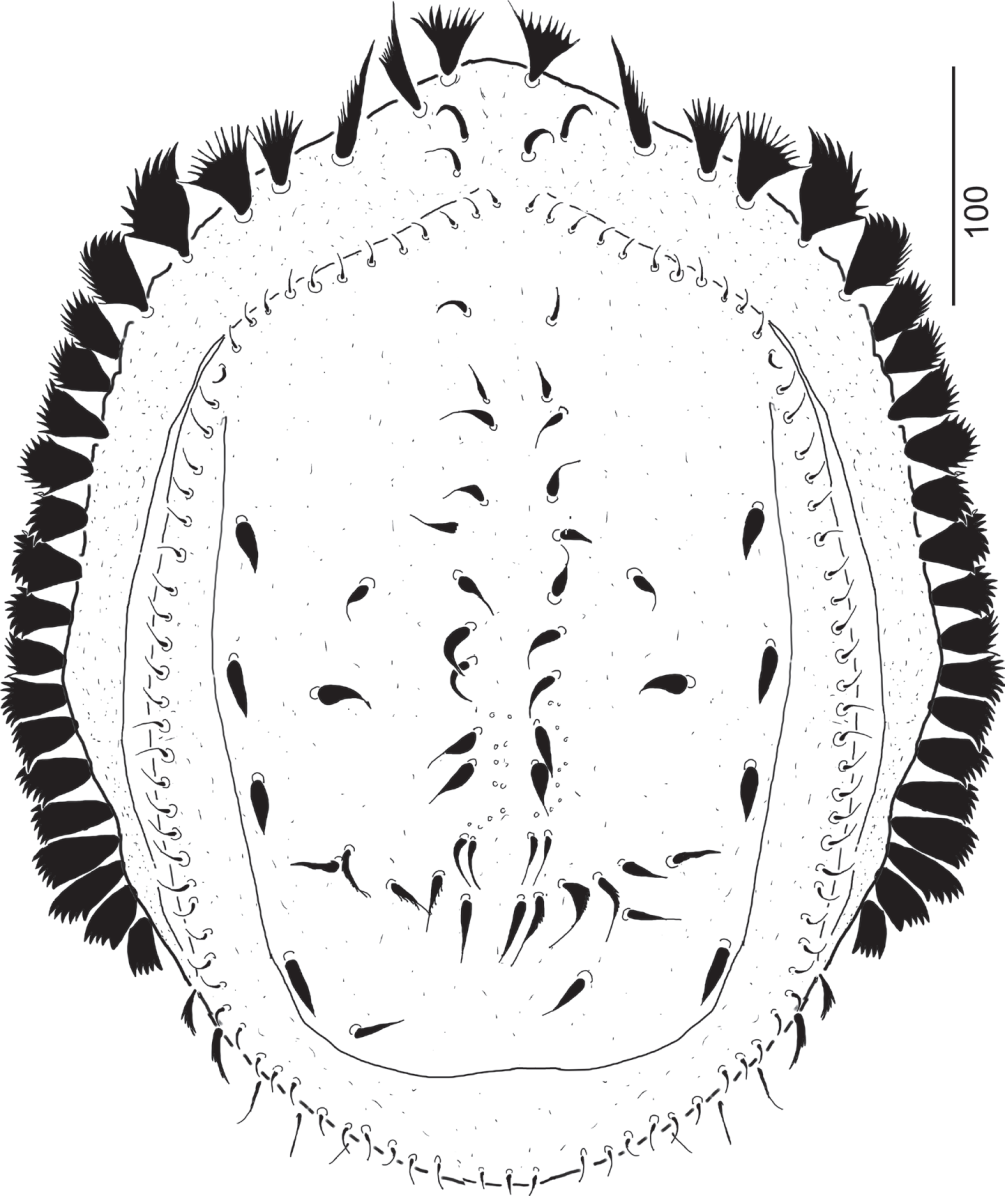
*Monstrobovellamancocapaci* sp. nov., holotype, female in dorsal view.

***Dorsal idiosoma*** (Fig. 2). Marginal and dorsal shields fused anterolaterally. Surface of dorsal shield without sculptural pattern, only some oval pits (*ca* 2 × 3) situated on posterocentral area. Margin of dorsal shield bears more than 45 pairs of short (*ca* 8–9) smooth and needle-like setae. Majority of other dorsal (more than 21 pairs) setae smooth and robust (*ca* 15–24 long). Three pairs of robust and marginally serrate (*ca* 22–24 long) setae situated on posterior part of dorsal shield. Marginal shield wide with more than 25 pairs of wide, phylliform (*ca* 35–45 long) and marginally-serrate setae. Two pairs of smooth and needle-like (*ca* 20–22 long) setae situated on anterior area of marginal shield. Pores and lyriform fissures not visible on dorsal- and marginal shields.

**Figure 3.**
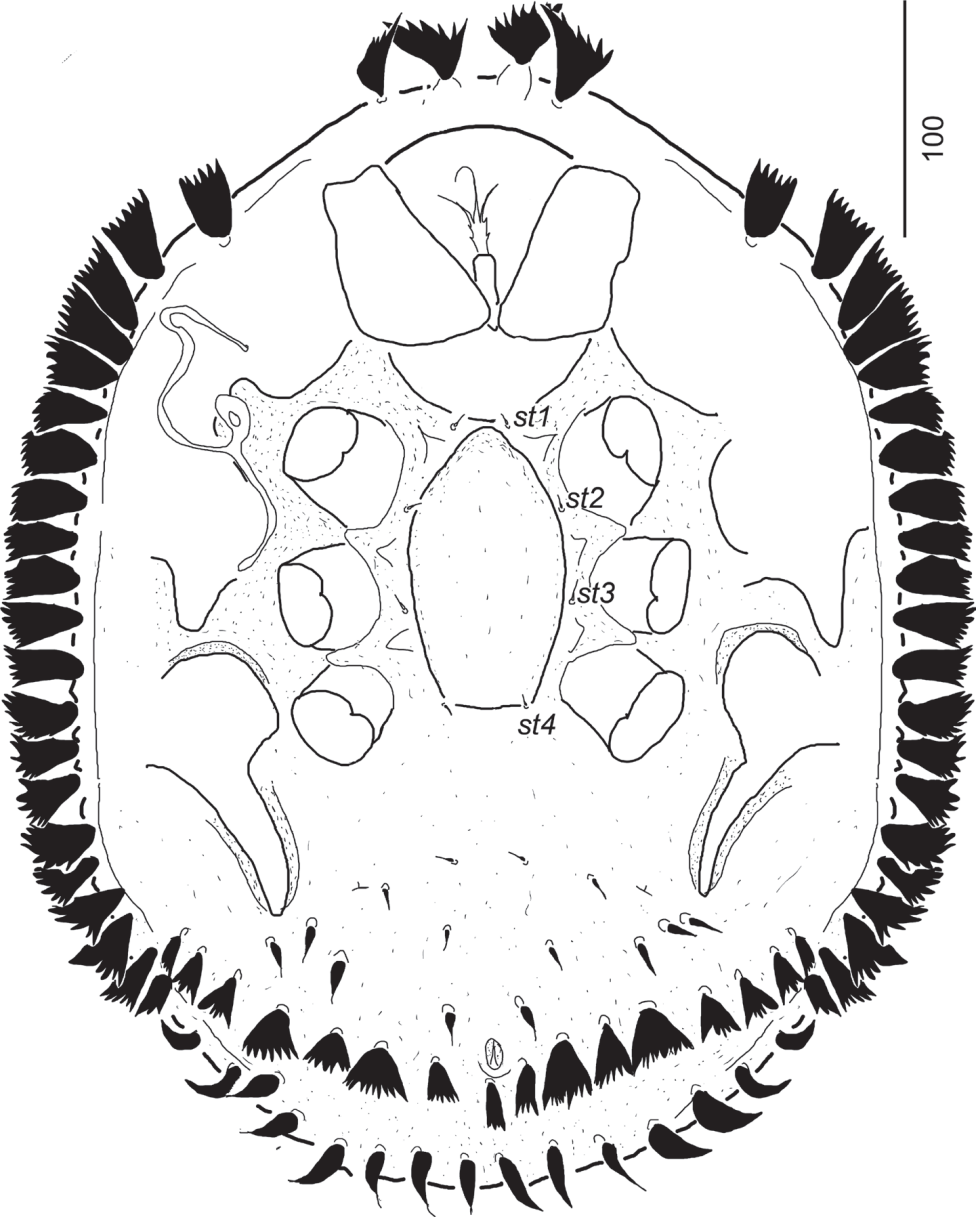
*Monstrobovellamancocapaci* sp. nov., holotype, female in ventral view.

***Ventral idiosoma*** (Fig. 3). Four pairs of sternal setae short (*ca* 7–9), needle-like and smooth. Setae *st1* inserted close to anterior margin of sternal shield, *st2* at mid-level of coxae II, *st3* at mid-level of coxae III, *st4* close to basal edge of genital shield. Sternal shield smooth, without any pores and lyrifissures. Three pairs of narrow and needle-like (*ca* 9–12 long) ventral setae situated between pedofossae IV. Three pairs of robust (*ca* 16–18 long) and spine-like ventral setae situated close to posterior end of pedofossae IV and one pair of robust and spine-like (*ca* 14–15) setae visible anterior to anal opening. Seven–eight pairs of wide, phylliform and marginally-serrate (*ca* 20–24 long) setae situated at level of anal opening and nine pairs of spine-like and very robust setae (*ca* 20–23 long) placed close to posterior margin of ventral idiosoma. One pair of lyriform fissure situated close to pedofossae IV. Ventral shield without ornamentation. Anal opening small and oval, *ca* 13–14 long and *ca* 7–8 wide.

Genital shield of female linguliform (*ca* 115–120 long and *ca* 60–65 wide) without sculptural pattern and without anterior process. Stigmata situated close to coxae II. Prestigmatid part of peritreme with two bends, poststigmatid part slightly curved. Pedofossae well developed, with smooth surface and separate furrow for tarsi IV.

Tritosternum with narrow base, tritosternal laciniae divided three branches, its basal part with two pairs of lateral spines (Fig. 3).

**Figure 4.**
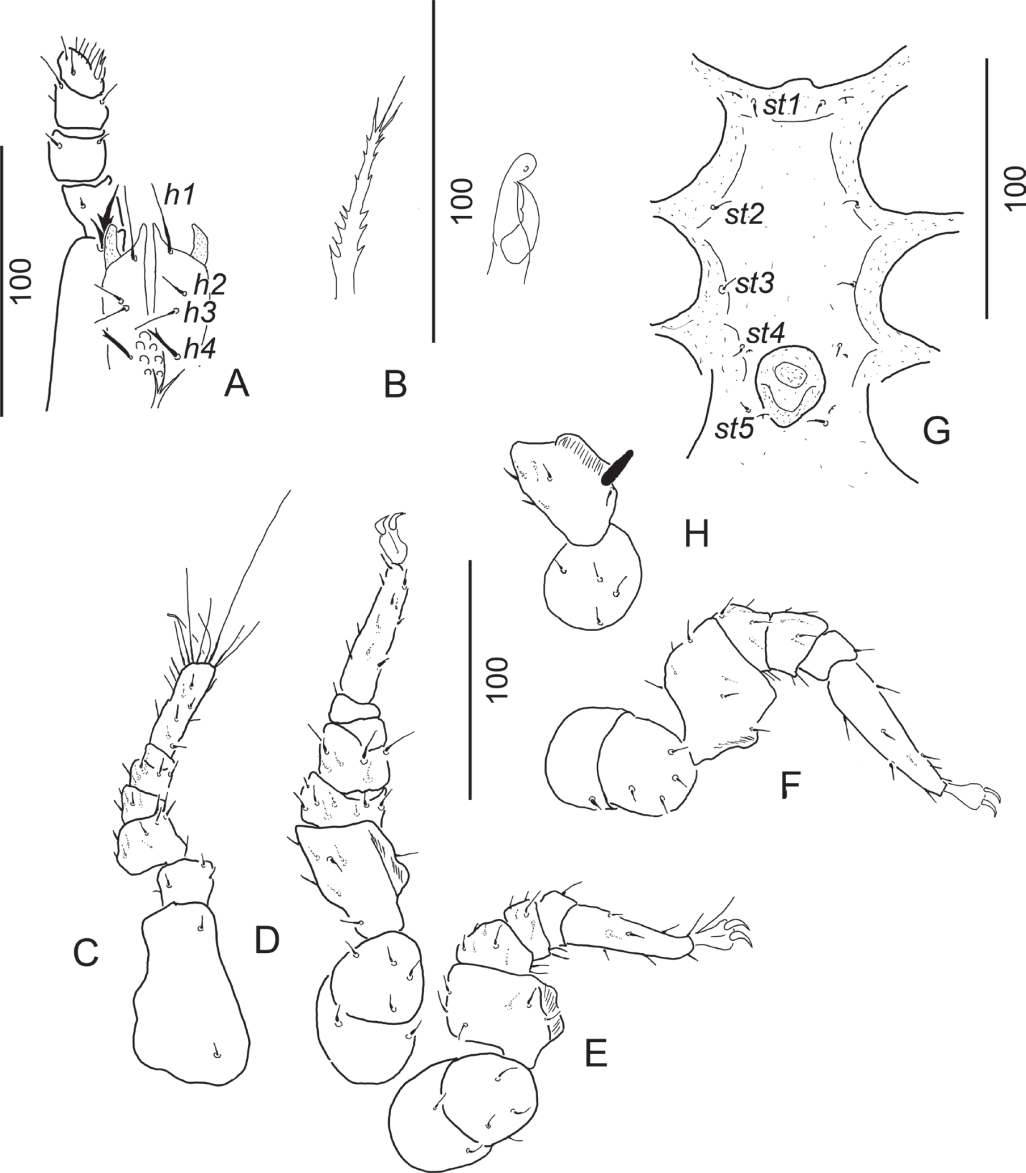
*Monstrobovellamancocapaci* sp. nov., holotype, female **A** gnathosoma and palp in ventral view **B** tritosternum in ventral view and chelicera in lateral view **C** leg I in ventrolateral view **D** leg II in ventrolateral view **E** leg III in ventrolateral view **F** leg IV in ventrolateral view **G** intercoxal area of male paratype **H** femur II of male paratype.

***Gnathosoma*** (Fig. 4A). Corniculi smooth and horn-like, internal malae smooth, shorter than corniculi. Hypostomal setae *h1*, *h2* and *h3* smooth and needle-like, *h1* long (*ca* 27–28), *h2* and *h3* short (*ca* 11–13), *h4* apically bifurcated and *ca* 14–15 long. Some rounded denticles situated between setae *h4*. Chelicerae with internal sclerotized nodes, fixed digit of chelicerae (*ca* 26–27) slightly longer than movable digit (*ca* 20–22) (Fig. 4B), only one small tooth situated on central part of movable digit. Palp trochanter setae *v1* longer (*ca* 19–22) and trifurcated, *v2* shorter (*ca* 13–15) and spine-like. Other setae on palp segments smooth. Palp apotele bifurcate (Fig. 4A). Epistome marginally serrate (Fig. 4B).

***Legs*.** Length of legs (from base of coxae to apex of tarsi): I 185–190, II 220–225, III 170–175, IV 200–205. Leg I without ambulacral claws; all setae on legs smooth and needle-like (Fig. 4C–F).

**Male** (*N* = 1). Body 590–630 long and 490–540 wide.

***Dorsal idiosoma*.** As in female.

***Ventral idiosoma*.** Intercoxal area, with sternal setae and genital shield, as in Figure 4G. Sternal setae *ca* 7–9 long, smooth and needle-like. Setae *st1* inserted close to anterior margin of sternal shield, *st2* at level of posterior margin of coxae II, *st3* at mid-level of coxae III, *st4* close to anterior margin of genital shield, *st5* close to basal margin of genital shield. Surface of sternal shield without any sculptural pattern. One pair of lyriform fissures situated close to anterior margin of sternal shield, other two pairs situated close to setae *st4* and *st5*. Genital shield rounded, slightly longer than wide (*ca* 35 × 25) and situated between coxae IV (Fig. G).

***Legs*.** Femora of leg II each with a long (*ca* 18) and robust ventral setae (Fig. 4H).

Other characters as in female.

**Developmental stages.** Unknown.

#### Etymology.

The species name is dedicated to Manco Cápac (Manco Qhapaq or Manku Qhapaq) the first king of the Kingdom Cuzcó and the first emperor of the Inca dynasty.

#### Remarks.

Only one *Monstrobovella* species (*M.faceta*) is known from the Neotropical region. *Monstrobovellafaceta* has T-shaped dorsal setae whose cross-bar part is bifurcated. These setae are spine-like and smooth in the new species. The setae on the caudal region of the dorsal shield are numerous, very long and marginally serrate in the case of *M.faceta*, and there are only three short, marginally-serrate setae in the new species. Several long and T-shaped setae are situated posterior to coxae IV on the ventral idiosoma; these setae are missing in the new species. There are some small oval pits on posterocentral part of dorsal shield in the new species, which are missing in *M.faceta*.

### ﻿Key to the known *Monstrobovella* species based on females

**Table:** 

1	Dorsal shield with some lateral furrows	**2**
–	Dorsal shield without furrows	**3**
2	Six long furrows situated on posterior part of dorsal shield	***M.crustosa***
–	Three long and three short furrows situated on posterior part of dorsal shield	***M.imadatei***
3	Dorsal setae T-shaped	4
–	Dorsal setae not T-shaped	***M.mancocapaci***
4	End of cross-bar of T-shaped setae bifurcated	**5**
–	End of cross-bar of T-shaped setae not bifurcated	**6**
5	Ventral setae long, longer than distance between ventral setae	***M.facetaoides***
–	Ventral setae short, distance between ventral setae longer than length of ventral setae	***M.faceta***
6	Dorsal shield with one pair of strongly sclerotized round-like depression on anterior region	***M.enodis***
–	Dorsal shield without strongly sclerotized round-like depressions	**7**
7	All of ventral setae bifurcated, simple setae absent	***M.incertaoides***
–	Ventral setae bifurcated or simple	***M.incerta***

## Supplementary Material

XML Treatment for Urodinychidae

XML Treatment for Elegansovella

XML Treatment for Elegansovella elegans

XML Treatment for Elegansovella pectinatasimilis

XML Treatment for Elegansovella serangensis

XML Treatment for Monstrobovella

XML Treatment for Monstrobovella crustosa

XML Treatment for Monstrobovella enodis

XML Treatment for Monstrobovella faceta

XML Treatment for Monstrobovella facetaoides

XML Treatment for Monstrobovella imadatei

XML Treatment for Monstrobovella incerta

XML Treatment for Monstrobovella incertaoides

XML Treatment for Monstrobovella mancocapaci

## ﻿Additional information

### Author ORCIDs

Jenő Kontschán https://orcid.org/0000-0001-8274-4238

Stefan Friedrich https://orcid.org/0000-0002-1668-4633

### Data availability

All of the data that support the findings of this study are available in the main text.
